# Swedish Movement and Massage Treatment

**Published:** 1889-08

**Authors:** 


**Swedish Movement and Massage Treatment.** By Prof. HARTVIG NISSEN, Director of the Swedish Health Institute, Washington, D. C. Philadelphia and London: F. A. Davis. 1889. Price $1.00.

The author begins his treatise by giving a short historical sketch of the gymnastic art as used in the treatment of various diseases. Bodily exercise, as a curative agent, was used in the earliest days; Æsculapius is said to have been the inventor, but Herodicus reduced it to a system, and made it a branch of medical science. It remained, however, for Ling, a Swede, in the early part of this century, to place "movement treatment" upon a scientific standpoint. The Ling system, or Swedish movement treatment, was soon adopted in many of the civilized countries, and Swedish movement institutes have been established in many of the larger cities.

The writer goes on to describe the various movements of the legs, arms, and trunk, as classified by Ling, their physiological and hygienic value, and their application to various diseases.

The chapters treating of the various movements are accompanied by numerous excellent illustrations, making the text at once clear and intelligible. Accompanying each description are recorded those cases in which the movement would be most likely to prove successful.

In the chapter on Diseases and their Treatment, the author takes up such constitutional diseases as chlorosis and anemia, neurasthenia, hysteria, and hypochondria, in each case giving explicit directions as to what movements are necessary, their duration, and frequency. The same plan is carried out in the treatment of local diseases, as anemia, and hyperemia of the brain and cord, paralyses, chorea, sciatica, diseases of the circulatory, respiratory, urinary, and motor systems. In some instances, the clinical history of a case is quoted, showing the result of the treatment.

This treatise, which claims to be the only manual of Swedish movement and massage treatment in the English language, is well worth the attention of those especially engaged in the massage treatment. The practitioner, or even specialist, would find little time for carrying out the details of the Ling system. The establishment of Swedish institutes in some of the larger cities would, however, enable him to give his patients the benefit of this mode of treatment.

The typographical part of the work is excellent; the binding, print, and illustrations are of the first quality, adding much to the value of such a manual. W. C. K.

